# Don’t forget the porpoise: acoustic monitoring reveals fine scale temporal variation between bottlenose dolphin and harbour porpoise in Cardigan Bay SAC

**DOI:** 10.1007/s00227-017-3081-5

**Published:** 2017-02-21

**Authors:** Hanna K. Nuuttila, Winnie Courtene-Jones, Sarah Baulch, Malene Simon, Peter G. H. Evans

**Affiliations:** 1 SEACAMS, Swansea University, Singleton Park, Swansea, SA2 8PP UK; 2 Sea Watch Foundation, Paragon House, New Quay, Ceredigion, SA45 9NR UK; 3 The Scottish Association for Marine Science (SAMS), Oban, Argyll, PA37 1QA UK; 4 University of York, York, YO10 5DD UK; 5 Environmental Investigation Agency (EIA), 62-63 Upper Street, N1 0NY London, UK; 6 Greenland Climate Research Center, Greenland Institute of Natural Resources, P.O. Box 570, Kivioq 2, 3900 Nuuk, Greenland; 7 Sea Watch Foundation, Ewyn y Don, Bull Bay, Amlwch, Isle of Anglesey, LL68 9SD UK

## Abstract

Populations of bottlenose dolphin and harbour porpoise inhabit Cardigan Bay, which was designated a Special Area of Conservation (SAC), with bottlenose dolphin listed as a primary feature for its conservation status. Understanding the abundance, distribution and habitat use of species is fundamental for conservation and the implementation of management. Bottlenose dolphin and harbour porpoise usage of feeding sites within Cardigan Bay SAC was examined using passive acoustic monitoring. Acoustic detections recorded with calibrated T-PODs (acoustic data loggers) indicated harbour porpoise to be present year round and in greater relative abundance than bottlenose dolphin. Fine-scale temporal partitioning between the species occurred at three levels: (1) seasonal differences, consistent between years, with porpoise detections peaking in winter months and dolphin detections in summer months; (2) diel variation, consistent across sites, seasons and years, with porpoise detections highest at night and dolphin detections highest shortly after sunrise; and (3) tidal variation was observed with peak dolphin detections occurring during ebb at the middle of the tidal cycle and before low tide, whereas harbour porpoise detections were highest at slack water, during and after high water with a secondary peak recorded during and after low water. General Additive Models (GAMs) were applied to better understand the effects of each covariate. The reported abundance and distribution of the two species, along with the temporal variation observed, have implications for the design and management of protected areas. Currently, in the UK, no SACs have been formally designated for harbour porpoise while three exist for bottlenose dolphins. Here, we demonstrate a need for increased protection and species-specific mitigation measures for harbour porpoise.

**Electronic supplementary material:**

The online version of this article (doi:10.1007/s00227-017-3081-5) contains supplementary material, which is available to authorized users.

## Introduction

In Cardigan Bay, West Wales, the two most common cetacean species, bottlenose dolphin (*Tursiops truncatus)* and harbour porpoise (*Phocoena phocoena*) are observed near shore throughout the year (Simon et al. 2010; Baines and Evans 2012). While bottlenose dolphin and harbour porpoise occur sympatrically, they are rarely observed in close proximity to one another (Pesante et al. 2008; Simon et al. 2010). The only reported associations between the two species in the UK are observations of aggressive behaviour by dolphins towards porpoises (Ross and Wilson 1996; Pesante et al. 2008; Norrman et al. 2015). In Wales (particularly Cardigan Bay), porpoise deaths caused by bottlenose dolphin attacks increased notably between 1991 and 2004, when peak mortality was recorded (Deaville and Jepson 2006; Penrose 2006, 2014). Within their population range, bottlenose dolphins and harbour porpoises show high variability in their distribution, with seasonal and diel fluctuations which typically correspond with the seasonal occurrence of prey species (Evans 1980; Northridge et al. 1995; Goodwin 2008; Sveegaard et al. 2012; Pirotta et al. 2014; Norrman et al. 2015). However, while studies of seasonal shifts in bottlenose dolphin distributions from spring to autumn have been relatively extensive (Wilson et al. 1997; Evans et al. 2003; Feingold and Evans 2014), less is known about their habitat use during the winter, due to the difficulty of conducting visual surveys in poor weather (Simon et al. 2010). For harbour porpoise populations, there is also uncertainty concerning their seasonal movements with little visual survey effort between November and March (Northridge et al. 1995; Evans et al. 2003). Furthermore, visual surveys cannot inform us of the nocturnal activity or diel changes in habitat use of the two species and it is only from acoustic studies that such data can be achieved (Bräger 1993; Philpott et al. 2007; Todd et al. 2009).

Acoustic monitoring is a useful tool for examining fine-scale changes in distribution, behaviour, and relative abundance of vocalising cetaceans. While visual surveys are limited by daylight, weather conditions and visibility, acoustic methods allow continuous monitoring, independent of weather and over the entire diel period, albeit often with limited spatial coverage. Static omni-directional click train detectors, known as T-PODs (Chelonia Ltd) have been widely used to study the relative abundance, behaviour, habitat use, and distribution of cetacean species in target areas (Carlström 2005; Philpott et al. 2007; Verfuß et al. 2007; Berrow et al. 2009; Rayment et al. 2009; Simon et al. 2010; Kyhn et al. 2012). T-PODs detect echolocation click trains, and both bottlenose dolphins and harbour porpoises produce these high frequency clicks for navigation as well as prey detection and discrimination. Echoes provide information on the range of targets and allow the animals to detect objects outside their visual range (Au et al. 2000). Porpoises emit only echolocation clicks and echolocate almost continuously (Akamatsu et al. 2007; Linnenschmidt et al. 2013), whilst dolphins produce clicks as well as whistles and burst-pulsed vocalisations for social communication (Au et al. 2000). Acoustic detection rates are thought to relate to the rate of occurrence of an echolocating species (Carstensen et al. 2006; Verfuß et al. 2007), and may give an indication of relative abundance (Simon et al. 2010; Kyhn et al. 2012). The characteristics of the echolocation clicks of both species are well documented (Au 1993, 1999; Villadsgaard et al. 2007). Despite some overlap, the echolocation signals of the two species have distinct characteristics and can be distinguished based on peak frequencies (frequency of maximum energy), bandwidth (the spread of energy across frequencies), and transmission beam width allowing both species to be monitored simultaneously.

Cetaceans face several threats from human activities, particularly from bycatch, but also from pollution, disturbance, prey depletion, and habitat degradation. Recent developments in marine renewable energy constructions, such as wind, wave and tidal devices, pose a potential threat to many cetacean populations particularly within the coastal zone (Carstensen et al. 2006; Brandt et al. 2011; Dähne et al. 2013). With the expansion of marine renewables across Europe, it is important that the potential impacts on cetaceans are considered, with mitigation and monitoring implemented early in the process (Wilson et al. 2007; Dolman and Simmonds 2010; Simmonds and Brown 2010), but also that these are considered together with existing threats, of which bycatch remains one of the greatest (Northridge et al. 2015). Bottlenose dolphins and harbour porpoises are both afforded protection under Annex II of the EU Habitats Directive due to their vulnerability from human activities in coastal seas. As a result, Special Areas of Conservation (SACs) have been established for both species to form a network of protected sites across Europe. Two of the three UK SACs for bottlenose dolphin are situated in Cardigan Bay (Wales), whereas the harbour porpoise is currently listed only as an additional feature for both sites with a grade D status of ‘insignificant presence’. Harbour porpoise have been reported in Cardigan Bay SAC year round (Simon et al. 2010) and the area of this study is in the process of being designated as a SAC (The West Wales Marine SAC) (Natural Resources Wales 2015).

Understanding the habitat preferences and fine scale distributions of the two species is important for conservation management, particularly ensuring that adequate protection is provided for both bottlenose dolphin and harbour porpoise populations. This study examined the spatio-temporal fluctuations in the distribution and occurrence of bottlenose dolphins and harbour porpoises within Cardigan Bay SAC with implications for future management. We used static passive acoustic monitoring and modelling to examine fine-scale seasonal, diel and tidal changes in the presence of dolphins and porpoises at selected hotspots.

## Materials and methods

### Study area

**Fig. 1:**
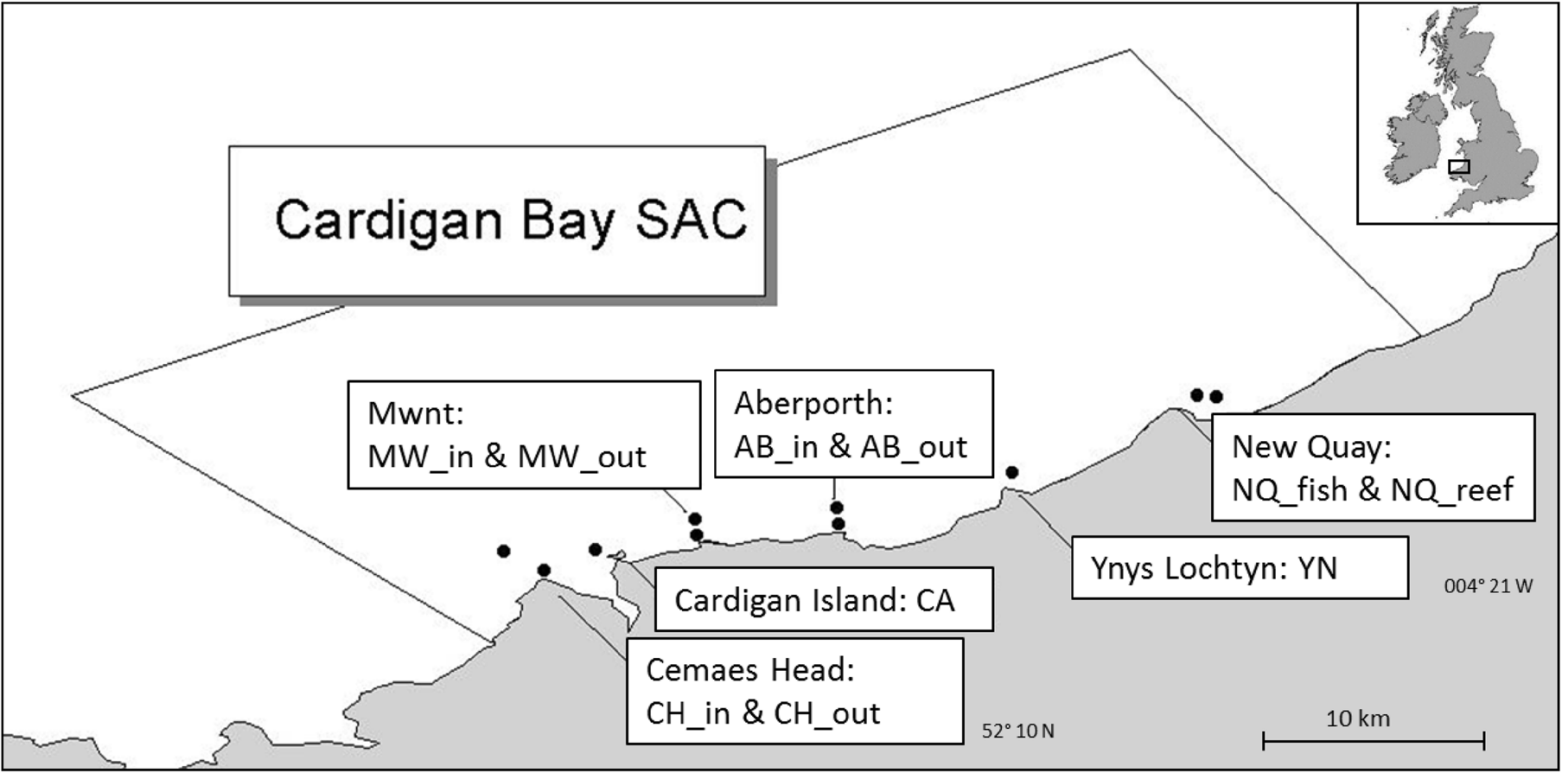
Map of study area in Cardigan Bay Special Area of Conservation (SAC). SAC boundaries are indicated by the grey lines. The points indicate T-POD deployment sites. ‘In’ stands for inshore location and ‘Out’ of offshore T-POD location

Cardigan Bay SAC is located in the south of Cardigan Bay, West Wales and covers an area of 1040 km^2^ (Fig. 1). The bay is exposed to the prevailing westerly and south-westerly winds and it has semi-diurnal tides with a mean spring tidal range of 4–5 m. The tidal currents are normally lower than 3.3 km/h flowing north during the flood, and south during the ebb (Evans 1995).

At three locations (Cemaes Head, Mwnt and Aberporth), two version 4 T-PODs were deployed, one inshore (approximately 300 m from the coast) (named ‘site name_in’) and one further offshore (800–1000 m from the coast), (named ‘site name_out’). In addition, a single T-POD was deployed at Ynys Lochtyn and Cardigan Island, and a further two in New Quay Bay (New Quay Reef and New Quay fish factory) (Fig. 1); all T-PODs were located at depths of 12–25 m. Ten T-PODs were deployed from April 2005 until February 2007, sampling all sites. From January to December 2008, six T-PODS were deployed at Aberporth inshore, Cardigan Island, Mwnt inshore, New Quay reef, New Quay fish factory and Ynys Lochtyn. During January to March 2009, T-PODs were deployed at all sites surveyed the previous year apart from New Quay fish factory (Table 1) with only single T-POD logging in Mwnt inshore between April and September 2009 and in Ynys Lochtyn between October and end of December 2009. Data were typically logged continuously for 5–6 weeks before being downloaded and the T-PODs re-deployed, although at times PODs would stop logging due to loss of battery life, memory card filling up, and entanglement in fishing gear or other unexplained reasons. As the work was conducted from small open boats, T-PODs had to be brought to shore for data download and gaps between deployments inevitably occurred due to weather and other logistical constraints (Table 1). The T-PODs were not rotated between sites and due to lack of funding, lost loggers could not be replaced by new ones.

**Table 1:**
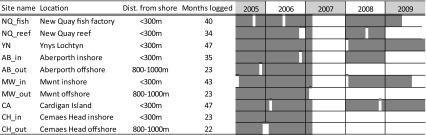
Summary of acoustic monitoring periods for each C-POD locations with the relevant abbreviated code names for the sites listed from North to South

White gaps denote periods of more than 2 weeks where data were not collected

### Data loggers

Echolocation signals were recorded using omni-directional, battery powered, static acoustic click detectors called T-PODs (http://www.chelonia.co.uk) which consist of a hydrophone, an amplifier, a number of band-pass filters, and a data logger with a timer in a hard plastic tube. These can be programmed to automatically log the time and duration of click trains from porpoises and dolphins, which fulfil several acoustic criteria set by the user such as click length (duration), frequency spectrum and intensity, and can be set to match the specific characteristics of echolocation clicks of the target species. The logger cycles through six adjustable frequency scans per minute and compares the output of a pair of band pass filters with adjustable bandwidth, one set to the click frequency of the species of interest and the other a reference frequency outside the target frequency (Verfuß et al. 2013). The energy picked up by the target filter must be a certain amount higher than the energy picked up by the reference filter to cause a registration of the presented sound.

The logger was designed specifically to log porpoise sonar signals as these are of a highly stereotypical nature and unique in being very short (50–150 µs) containing very little energy below 100 kHz. The main part of the energy is in a narrow band of 120–150 kHz, which makes these signals ideal for automatic detection as most other noise in the sea typically contains energy over a wider frequency range, is of longer duration, and has peak energy at much lower frequencies. The only noise source of similar frequency range, the boat echo sounder, produces clicks of regular pattern, whereas porpoise echolocation click pattern has decreasing and increasing inter-click intervals (Teilmann and Carstensen 2012). Similarly to porpoise clicks, dolphin clicks can also be detected by the T-POD (Philpott et al. 2007). Dolphins tend to emit louder clicks and can therefore be detected at much larger ranges; however, they have a higher likelihood of not emitting echolocation clicks and therefore have a lower detection probability of being detected than porpoises.

Three of the six scans were set to detect bottlenose dolphin clicks with target filter at 50 kHz and reference filter at 70 kHz (Philpott et al. 2007; Bailey et al. 2010; Simon et al. 2010). The remaining three scans were set for harbour porpoises with target filter frequency of 130 kHz and a reference filter at 92 kHz (Philpott et al. 2007; Simon et al. 2010). No other cetacean species occur, except as vagrants, in the area (Pesante et al. 2008; Baines and Evans 2012).

To account for the variability and to ensure the inter-comparability of the T-PODs (Kyhn et al. 2008), they were calibrated before undertaking any research. Since the dolphins emit clicks at overlapping frequencies to the harbour porpoises, although with energy across a much broader bandwidth and of shorter duration, the possibility of recording dolphin clicks on porpoise scans needed to be accounted for. For this study, prior to deployment, Simon et al. (2010) therefore conducted an extensive test-tank calibration experiment at the German Oceanographic Museum, Stralsund, Germany (Verfuß et al. 2008). The experiments were carried out to determine the most suitable ‘bandwidth/selectivity’ as well as the ‘threshold/minimum intensity’ settings for each T-POD where a majority of dolphin clicks would no longer be registered in the porpoise scan and vice versa. The laboratory calibrations were also validated with a field calibration to further ensure the inter-comparability of the T-PODs (Simon et al. 2010).

### Data analysis

The study yielded data from 1220 days between April 2005 and December 2009 with a gap in data collection from March to December in 2007. Acoustic data were downloaded with the software T-POD.exe (version 8.17; Chelonia Ltd, Cornwall, UK, http://www.chelonia.co.uk), which uses an in-built train detection algorithm to filter through the raw click data, identifies cetacean click trains, and estimates their probability of arising by chance from a non-train producing source (such as rain or a boat propeller). This probability, *p* is determined by a Poisson distribution of the prevailing rate of arrival of clicks, the size of the interval between each click and the regularity of the trains. The probability of an entire identified train arising by chance from random sources will be the product of successive *p* values. The software then assigns the click trains to categories by species and their probability of being from cetacean origin. To minimise the risk of including a significant number of false positive detections, only data deemed by the algorithm as highly likely to be of cetacean origin were used (the ‘cet high’ and ‘cet low’ categories) while the rest of the click trains (the ‘doubtful’, ‘very doubtful’ and ‘boat sonar’ categories) were excluded (Carstensen et al. 2006; Tougaard et al. 2009). Although this meant that some real detections were missed as a consequence, this was considered more desirable than to include potential false positive detections in the analysis. Since the study site yielded an excessively large amount of detections for both species, no further false positive analysis was conducted for this dataset. To describe the presence of dolphins and porpoises at each site, we exported the number of detection positive minutes (DPM) per hour for both species (Simon et al. 2010). The final dataset consisted of over 169 620 h of data, including 45 974 min of dolphin detections and 165 204 min of porpoise detections.

Sunrise and sunset times for each day of each year were obtained from the U.S. Naval Observatory (Astronomical Applications Dept. Nautical Twilight Times, http://aa.usno.navy.mil/). Differences between daylight times for different T-POD locations were minimal (less than 2 min), and therefore times for the central location, Aberporth were used for all locations. Tidal data were obtained from Ceredigion County Council, with appropriate adjustments made for each site. Seasons were classified as ‘Winter’ (December–February), ‘Spring’ (March–May), ‘Summer’ (June–August) and ‘Autumn’ (September–November). The variables selected for analyses were year, month, hour of the day, time from low water, time from sunset, maximum tidal range, and presence of another cetacean species. Presence of another cetacean was logged if detection positive minutes were recorded for both species within the same 1-h period.

Statistical analysis was conducted with Generalised Additive Models (GAM) and Generalised Additive Mixed Models (GAMM) using log link and negative binomial distribution to account for overdispersion. The response variable was the total number of DPM logged in an hour. This yields counts of porpoise and dolphin detections for each hour recorded. Prior to running the models, data were tested for collinearity using the *vif* function, with 2 as cut-off value. Autocorrelation of the response variable was assessed using ACF plots for each location with 0.2 as the autocorrelation threshold. No strong evidence of autocorrelation was found although there was slight elevation in ACF valued for the first lags in the porpoise data and the residual values in the diagnostic plots showed some patterns. Models were therefore run both with and without AR1 structure which allows for autocorrelation within the first time lag for non-normally distributed variables and longitudinal data, and can assist in modelling temporally and spatially auto-correlated data. No change was found in diagnostic plots for residuals when including AR1 error. None of the covariates were found to be collinear and so all selected variables were retained in the model. Variables were sequentially removed from the model and selection was based on Akaike’s Information Criterion (AIC); adjusted *R* ^2^ values with diagnostic plots were inspected to assess overall model fit. Location was included as a factor variable as it was not possible to include it as a random variable in a mixed effects GAMM model due to the size of the dataset. To evaluate whether this would mask the effect of some covariates, models were also run for each site separately. All statistical analyses were carried out in R version 3.12 (R Development Core Team 2013) and R Studio version 0.98.1091 (RStudio Team 2015) using R packages *mgcv, gamm, nlme* and *car*.

## Results

### GAM modelling

**Fig. 2:**
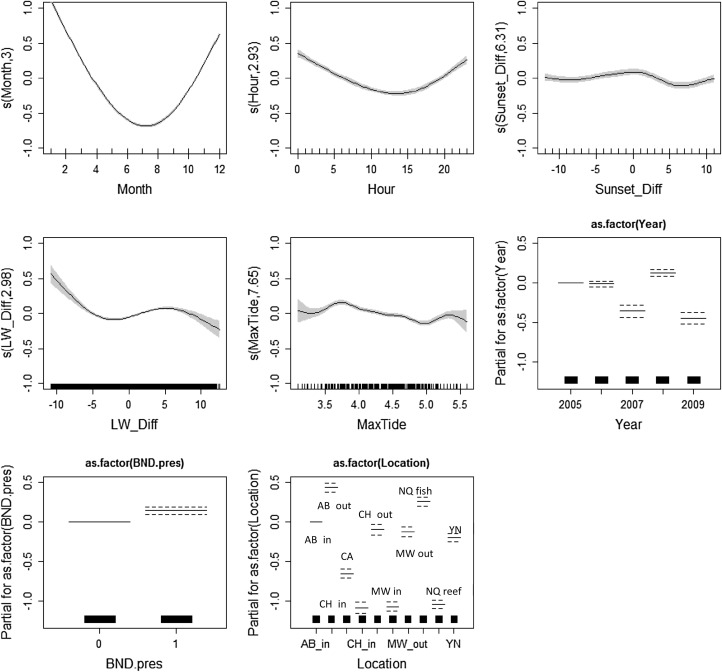
Fitted relationships for the harbour porpoise model based on GAM standard errors. The *grey areas* around the relationship are the 95% confidence intervals for each smooth covariate. For factor variables this is represented by *dotted lines. Y* axis shows the partial residuals for each model covariate generated by regressing the response on the other covariates. A larger *y* axis indicates a more important covariate

**Fig. 3:**
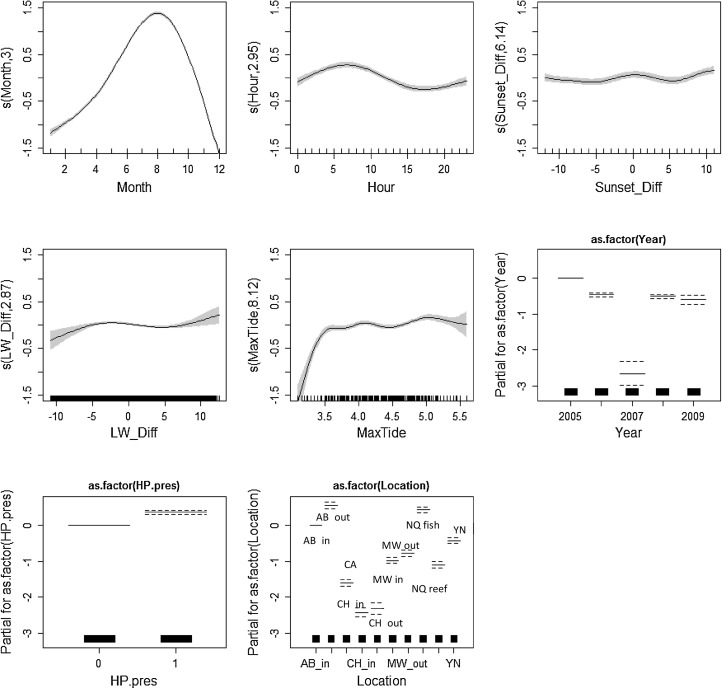
Fitted relationships for the bottlenose dolphin model based on GAM standard errors. The *grey areas* around the relationship are the 95% confidence intervals for each smooth covariate. For factor variables this is represented by *dotted lines. Y* axis shows the partial residuals for each model covariate generated by regressing the response on the other covariates. A larger *y* axis indicates a more important covariate

**Fig. 4:**
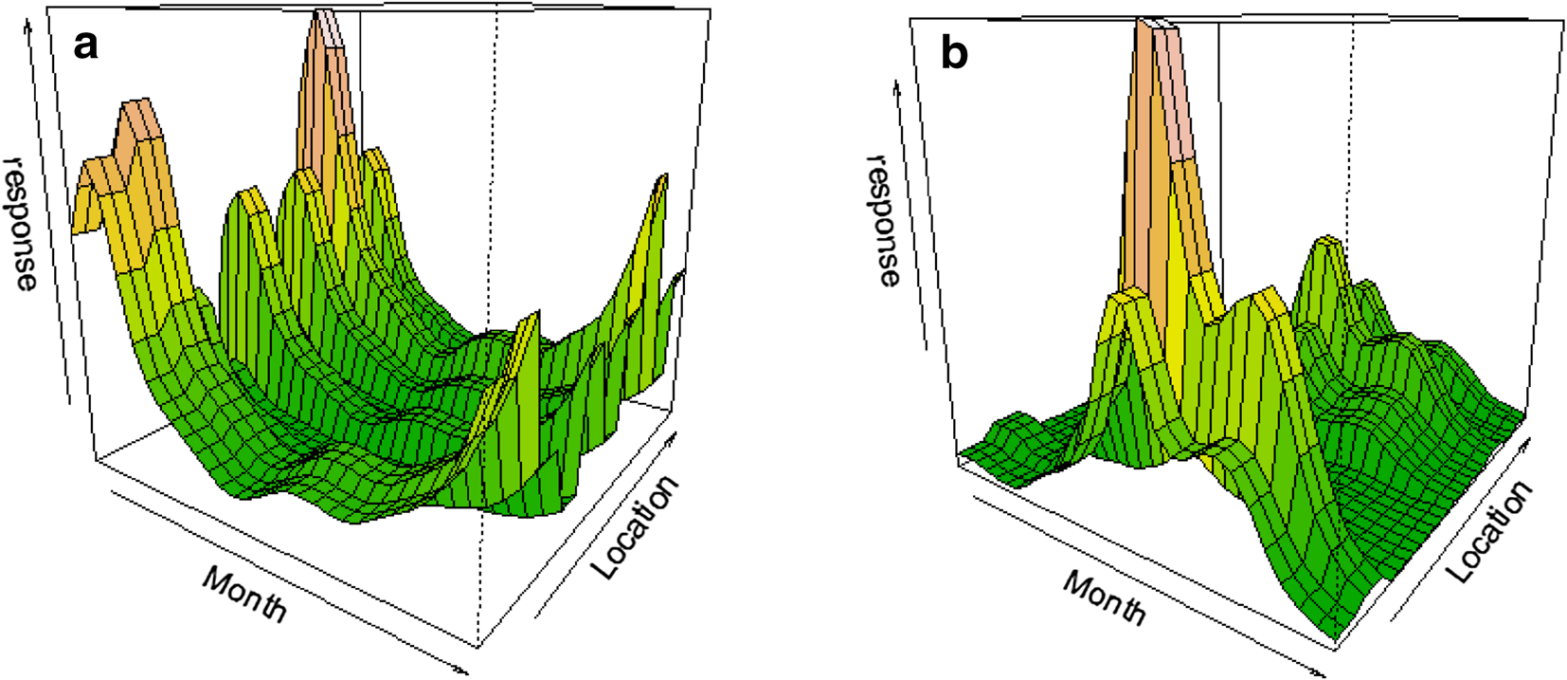
3D representation of the effect of month and location on the response scale for each site for both harbour porpoise (**a**) and bottlenose dolphins (**b**)

The final model for both species included year, month, hour, time from sunset, max tide, time from low water, location and other species’ presence. No interaction terms were included in the models as these had only negligible effect on the model results. Model results for smooth plots for porpoises are depicted in Fig. 2 and for dolphins in Fig. 3, and summaries of the results are listed in the Electronic Supplement. Location and Month were the most important covariates for both species based on AIC criteria the adjusted-*R* ^2^ values (Table 2). Year was the third most important variable for both species although the lack of data in 2007 is evident in the smooth graphs for the dolphin data. Similarly, for porpoises the difference in effect sizes for years 2007 and 2009 compared to 2005, 2006 and 2008 is obvious and points to the gaps the data collection. All the other covariates had significant effects for both species and models but their ranking based on the AIC criteria and the adjusted-*R* ^2^ values varied. Examining the covariate Location showed that deployment site had equal effect within both porpoise and dolphin models (Fig. 4). Aberporth inshore and offshore, Cemaes Head inshore, Mwnt offshore and Ynys Lochtyn had nearly equal effect sizes, as did New Quay Reef, Mwnt inshore and Cemaes Head inshore. Separate from both were New Quay fish factory and Cardigan Island.

**Table 2:** The relative importance of each model covariate in explaining the observed activity patterns

BND covariate	% Decrease in adj. *R* ^*2*^	Adj. *R* ^2^ rank	Change in AIC	AIC rank	HP covariate	% Decrease in adj. *R* ^2^	Adj. *R* ^2^ rank	Change in AIC	AIC rank
Month	29.45590994	2	5920.4	2	Month	38	2	5944.5	2
Year	−7.879924953	8	619.1	3	Year	5	3	314.8	3
Hour	0.562851782	5	71.3	6	Hour	2	4	198	4
Sunset	1.500938086	4	35.2	7	Sunset	1	5	35.9	6
Low water	−0.375234522	6	14.4	8	Low water	−1	7	96.2	5
Maxtide	−2.063789869	7	129.7	5	Maxtide	−2	8	157	4
HP	5.253283302	3	214.6	4	BND	0	6	29.2	5
Location	29.08067542	1	5922.8	1	Location	50	1	5930.2	1

Values are derived from models where the listed covariate has been removed. Covariates are ranked from 1, the highest importance, to 6 (BND) or 7 (HP), the lowest importance

### Final model for each site separately

The effect of Location on DPM/h was larger for porpoise than for dolphins, although Month was still the most significant covariate for each site. Max Tide made less of a difference at New Quay fish factory than other sites; Hour of the day and Time from Sunset had relatively little effect at Cemaes Head inshore site and Time from Sunset at New Quay Reef had little effect compared to other variables. Porpoise presence had the least effect on the model at all sites apart from Cemaes Head where Hour and Time from Sunset both had less of an effect.

For porpoise data, the effect of smooth variables (month, hour, time from sunset, time from low water, max tide) was almost identical to the full dataset at all ten locations, apart from Cemaes Head inshore where Time from Sunset had no effect on the model. The effect of Year was not significant at Cemaes Head inshore or Aberporth offshore locations. Dolphin presence was significant in all but the Mwnt inshore location.

### Spatial variation

**Fig. 5:**
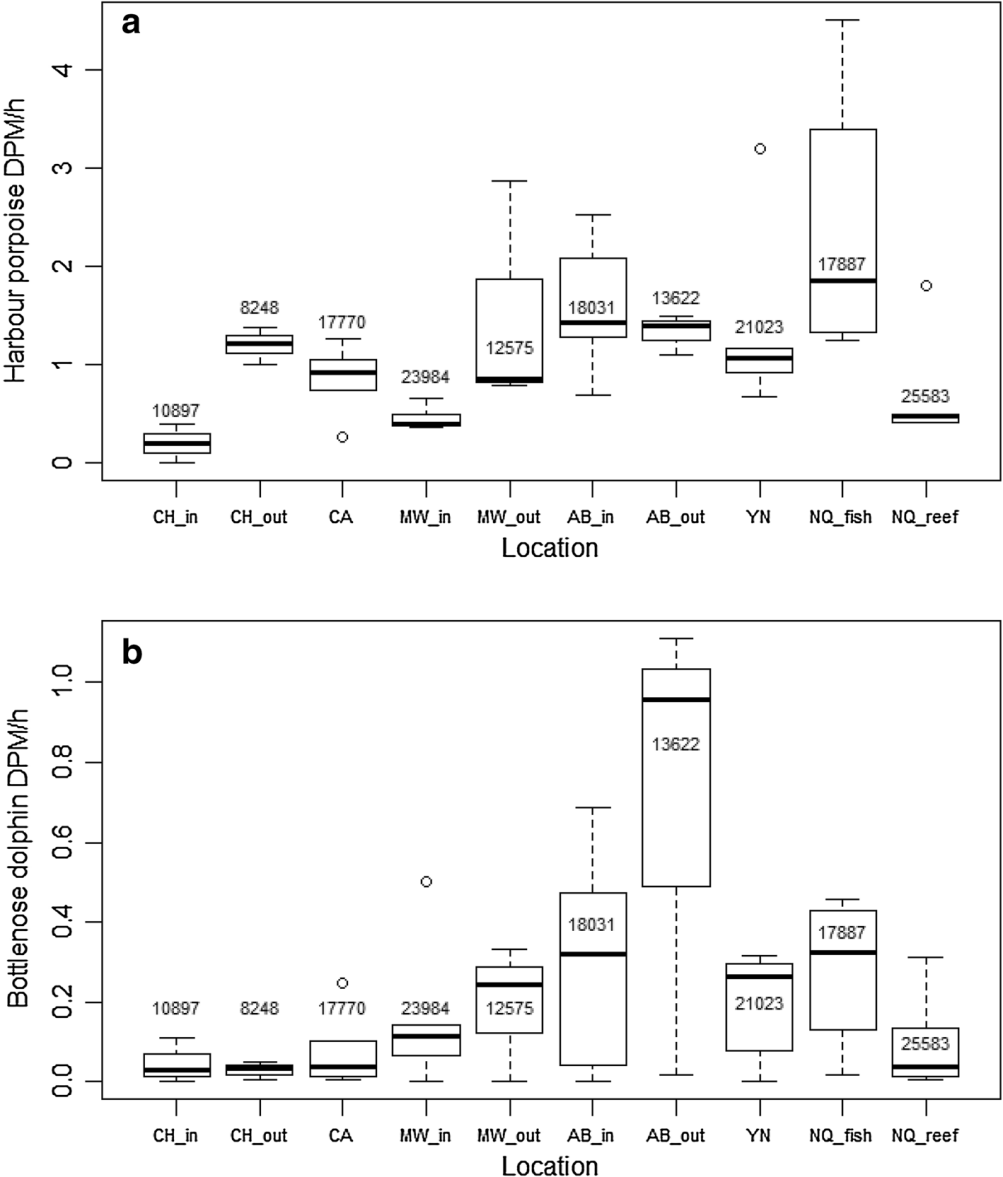
Mean Detection Positive Minutes (DPM) per hour for (**a**) harbour porpoise and (**b**) bottlenose dolphin at each monitoring location in Cardigan Bay SAC with sample sizes shown for each location. Note different scales for *Y* axes. The interquartile range is shown by the *box* with the *thick black line* representing the calculated yearly mean value for each location; *circles* show data points lying outside the 95% confidence limits. See Fig. 1 for locations

Site use within Cardigan Bay SAC varies widely between bottlenose dolphins and harbour porpoises. However, certain locations, such as Aberporth, are important for both species. Highest detections (DPMs) per hour for harbour porpoise were recorded at Aberporth inshore site, while the southern and westernmost sites, Cemaes Head and Mwnt consistently recorded the lowest harbour porpoise DPMs/h across the years 2005–2009. New Quay fish factory was observed to have an increase in harbour porpoise DPMs/h across years. Bottlenose dolphins show a preference for both Aberporth sites with the highest detections recorded at these locations, while Cemaes Head offshore had the lowest DPMs/h of all sample locations for dolphins (Fig. 5).

### Inter-annual and seasonal variation

**Fig. 6:**
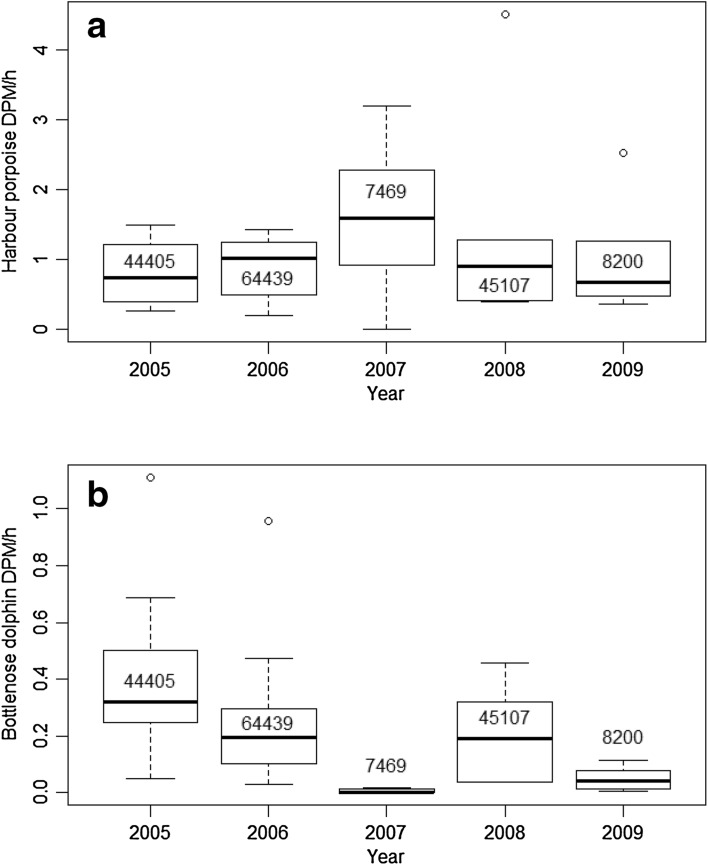
Mean inter-annual Detection Positive Minutes (DPM) per hour for (**a**) harbour porpoise and (**b**) bottlenose dolphin with sample sizes shown for each year. The interquartile range is denoted by the *box* with the *thick black line* depicting the mean values for each year; *circles* show data points outside the 95% confidence limit. Note 2007 data is biased as data was only collected in January and February and in 2008 and 2009 only six T-PODs were deployed—see Table 1

Detections were consistently higher for harbour porpoise than bottlenose dolphins across all years. Detections for harbour porpoise were relatively stable across years except for 2007. Bottlenose dolphins exhibited a more varied pattern: DPM were similar during 2005, 2006, 2008, with lower detections in 2007 and 2009 (Fig. 6), even though data collection started in spring of 2005. However, in 2007, data were collected only in winter (January and February), which would explain the relatively high porpoise and low dolphin detections.

**Fig. 7:**
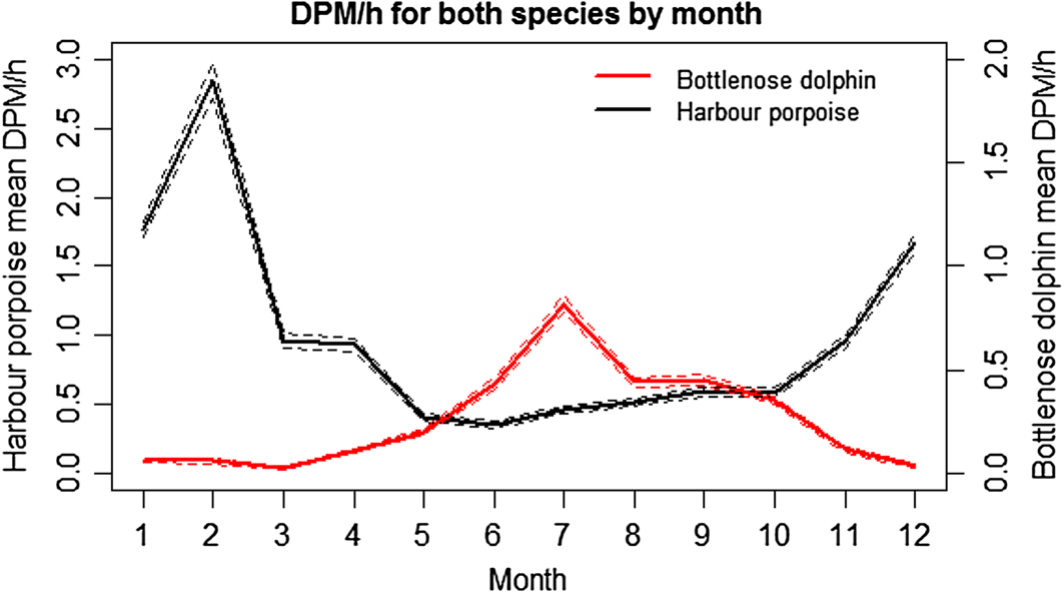
Monthly variation in mean harbour porpoise and bottlenose dolphin Detection Positive Minutes (DPM) per hour. *Dashed line* depicts the 95% confidence interval around means. Note the different scales for the *y* axis

A clear seasonal pattern is observed by both species for each of the years well sampled. Harbour porpoise detections are highest across the winter months December-February, with a clear peak in DPM recorded in February, and the lowest detections occurring during the summer months, June–August. Bottlenose dolphins display the opposite pattern, with detection rates highest during summer months, June–August, peaking during July and then falling throughout the subsequent months, reaching the lowest mean DPM during winter December-February (Fig. 7).

### Diel variation

**Fig. 8:**
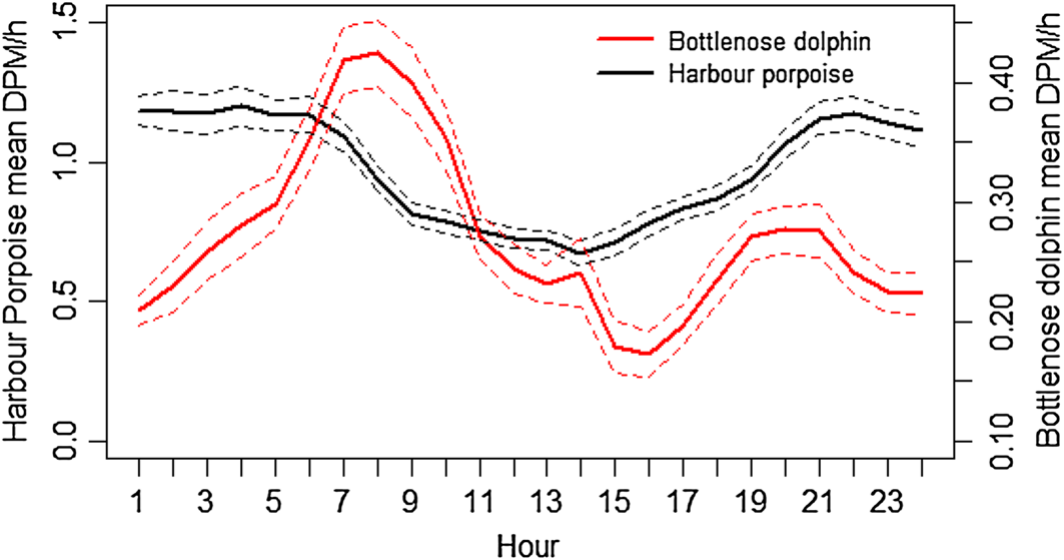
Mean Detection Positive Minutes (DPM) per hour for harbour porpoise and bottlenose dolphin across the diel cycle. *Dashed line* depicts the 95% confidence interval around mean values. Note the different scales for the *y* axis

Overall mean DPM was higher for harbour porpoise than bottlenose dolphin throughout the diel cycle. Harbour porpoise detections showed a clear pattern; the highest mean detections occurred during night-time, from 21:00–06:00 h, which corresponded with the lowest detections of bottlenose dolphins. Harbour porpoise mean detections decreased dramatically just after sunrise and remained low throughout daylight hours until approximately 19:00 h, the average time of sunset, when detection rates increased sharply. Dolphin detections were relatively low throughout the night but increased just prior to dawn, peaking after sunrise at approximately 08:00 h. Throughout the rest of the day, detection rates decreased progressively until 16:00 h when there was a second increase, coinciding with sunset (18:00–21:00 h) (Fig. 8).

### Tidal variation

**Fig. 9:**
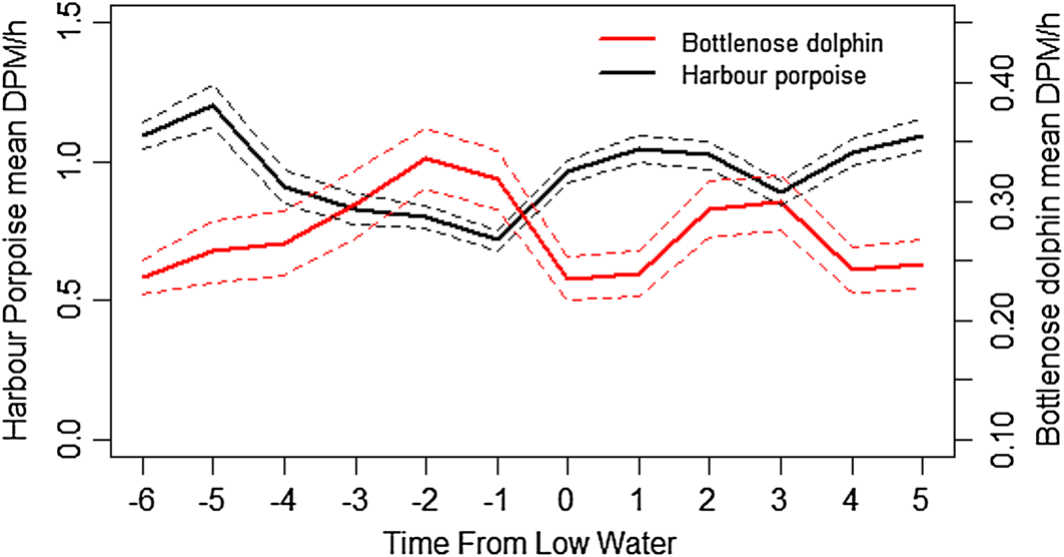
Mean detection positive minutes (DPM) per hour of both species across the tidal cycle. Ebb = −6 to 0 h before low water, flood = 0 to 6 h after low water. *Dashed line* depicts the 95% confidence interval around mean values. Note the different scales for the *y* axis

Detections of both species varied across the tidal cycle; and displayed an opposite pattern. Bottlenose dolphin detections showed a clear peak in DPM during the middle of the ebb phase before low water, and a secondary peak during the middle of the flood phase, when the tidal flow is at its strongest. Trends for harbour porpoise relating to the tidal cycles were less clear than for dolphins. Harbour porpoise DPMs were highest during and just after high water, but with a secondary peak during and just after low water, both of which are on or close to slack water times, when the tidal flow is at its lowest (Fig. 9).

### Interspecific interactions

There was small but significant positive effect of dolphin presence affecting the presence of harbour porpoise and vice versa despite initial data analyses showing a negative relationship between dolphin presence and porpoise detections. This seems to reflect a difference between the apparent relationship between the variables and the partial relationship emerging from the model.

## Discussion

In this study, long-term acoustic monitoring was used to show that both bottlenose dolphins and harbour porpoises are present in Cardigan Bay SAC year round, supporting previous work by Simon et al. (2010). Deploying and retrieving static acoustic monitoring gear poses many challenges and if only small inshore vessels can be used for deployment and retrieval, gaps in data collection are unavoidable due to weather and other logistical constraints. This can lead to an unbalanced data set, which should be considered in any analysis. Here, the longevity of the study enabled inter-annual comparison although the gaps had to be taken into consideration when interpreting the data.

The results demonstrate clear partitioning between bottlenose dolphin and harbour porpoise, occurring at a various levels, with distinct site, seasonal, diel and tidal differences observed between the two species. Generalised Additive Models (GAM) were applied to understand the effect and relative importance of these covariates for each species. Month and location were shown to have the greatest effect on both species’ presence (Table 2), highlighting the clear seasonal variations observed, which have been documented in previous studies elsewhere (Verfuß et al. 2007; Fury and Harrison 2011). It is possible that site-specific differences in detections might reflect the T-PODs’ internal sensitivities but the acoustic data presented here support site-specific differences identified by visual surveys (Pesante et al. 2008). The model fits were assessed using model diagnostics plots. For harbour porpoise data, no distinct patterns of residuals were seen. For dolphin data, there seemed to be some pattern in residuals indicating potential non-independence of residual errors, although including an auto-correlation term did not improve the residual spread.

Both monitoring sites at Aberporth were used frequently by both species. For bottlenose dolphins, Aberporth sites had double the number of detections than any other site. New Quay fish factory site was identified as the overall most popular site for harbour porpoise, although in summer the highest porpoise DPM were recorded also at Aberporth (Fig. 2). Organic waste disposal by the factory probably explains the year round popularity of New Quay fish factory. It is less clear what features make the Aberporth site important for both species, although boat surveys have also identified it as a favoured feeding location for dolphins within the SAC (Pesante et al. 2008; Feingold and Evans 2014), whilst a study of these same sites found that dolphin sightings had increased at Aberporth and Mwnt over the period from 1995 to 2007 (Pierpoint et al. 2009).

Detection rates of dolphins and porpoises varied markedly between seasons, with an inverse relationship observed. Detections of dolphins were highest during the summer while for porpoises they were highest during the winter months, in accordance with trends from visual surveys and initial acoustic analyses (Pesante et al. 2008; Simon et al. 2010). As suggested by studies on porpoises in German waters (Schaffeld et al. 2016) these seasonal patterns of occurrence of the two species may be driven by prey availability and the fish species targeted, although requirements for suitable environmental conditions for calving may also play a part. Although the prey preferences of the two species and the prey distribution and movements are not fully known, the late summer peak in numbers of dolphins seems to correspond with seasonal concentrations of migratory fish such as mackerel (*Scomber scombrus*) and salmonids ICES (2011) in Cardigan Bay (Pesante et al. 2008). By contrast, during winter the dolphins tend to disperse over wider areas and move further north, occurring in significantly larger groups. Here, they appear to be feeding on shoaling fish such as herring (*Clupea harengus*) and whiting (*Merlangius merlangius*), as revealed from fish catches close by (Pesante et al. 2008). The summer increase in dolphin detections also coincides with their calving season, which extends from May–September, peaking in July and August (Feingold and Evans 2014). The regular presence of prey and its shallow depth make inshore Cardigan Bay a favourable calving area. Similar seasonal increases in bottlenose dolphin presence are seen in the inner Moray Firth, coinciding with the seasonal migration of anadromous fish and the calving season for the species there (Wilson et al. 1997; Hastie et al. 2003); however, salmonids may not play such an important role in the diet of dolphins in Cardigan Bay, instead they have been observed catching seabass (*Dicentrarchus labrax*) in the area. Similarly, the reduction in relative abundance of dolphins in winter is likely to be driven by changes in prey availability. Although the prey of harbour porpoises overlaps that of bottlenose dolphins (Santos and Pierce 2003; Spitz et al. 2006), porpoises show markedly different seasonal variation in occurrence, with highest detection rates at these sites in winter. Both species prey on sandeels (*Ammodytidae*), cod (*Gadus morhua*), whiting, sprat (*Sprattus sprattus*), Atlantic herring, mackerel and a variety of flatfish, although dolphins also feed on salmonids, sea bass and grey mullet (*Chelon labrosus*) (Santos et al. 2001; Santos and Pierce 2003). In the Bay of Biscay, Spitz et al. (2006) found that bottlenose dolphins and harbour porpoise showed partial dietary overlap within their prey profile, foraging habitats, prey species and size range. However, differences in seasonal distributions of the two species suggest different prey and prey size preferences (cf. Rae 1965, 1973). Increases in porpoise occurrence in winter may coincide with concentrations of herring, typically an important prey species in areas where it occurs commonly (Recchia and Read 1989; Gannon et al. 1998; Börjesson et al. 2003; Sveegaard et al. 2012), although there is no evidence for this in Cardigan Bay. It might be that the increase in harbour porpoise detections during winter would be due to the relaxation of inter-specific interactions between the two species. It should also be noted that porpoise detections by an acoustic data logger can be affected by the location of the device in the water column (Sostres Alonso and Nuuttila 2015). Here, the T-PODs were moored close to the seabed and it is possible that some clicks emitted close to the surface could have been missed. Another factor which might influence the seasonal pattern of these species is the presence of recreational vessels and commercial fishing boats and wildlife watching boats which use the area mainly from April to October, and may have an impact (Dyndo et al. 2015; Pirotta et al. 2015). Interestingly, it is the dolphin detections that are at their highest during the peak recreational boating season, despite being the main target of the wildlife watching activities. Porpoise detections on the other hand decrease during the summer period, although there is no direct evidence to suggest that this is due to boating activities.

Acoustic detections of bottlenose dolphin and harbour porpoises varied across the diel cycle and showed the opposite trend to one another. Porpoise detections were most abundant at night whereas dolphins were mostly detected during the day. Higher rates of echolocation at night have been documented in harbour porpoises (Cox et al. 2001; Carstensen et al. 2006; Todd et al. 2009) and may compensate for loss of visual cues (Carlström 2005). However, if this was the sole explanation for the increase, we would expect a similar increase in echolocation rates for both species in response to low light, which is not the case, suggesting potential foraging activity of porpoise during the hours of darkness. It is more likely that increase in porpoise detections at night-time is due to increased foraging activity at night (Todd et al. 2009; Schaffeld et al. 2016).

Tidal relationships are well known in both porpoises and dolphins (Gregory and Rowden 2001; Mendes et al. 2002; Pierpoint 2008; Marubini et al. 2009; Embling et al. 2010; Fury and Harrison 2011; Isojunno et al. 2012). Here, we document different patterns between the two species across the tidal cycle: bottlenose dolphins showed a peak in acoustic detections at times when the average tidal flow is at its strongest; conversely, harbour porpoise detections were greatest during times close to slack water, when the tidal flow is at its lowest. Isojunno et al. (2012) suggested harbour porpoise use a range of current regimes, which enhance relative vorticity and concentrate prey in available high quality patches (Borges and Evans 1997).

Based on initial data analysis and graphic examination of the opposite trends in the two species, the presence of one species was expected to negatively affect the presence of the other, but the opposite was the case in this dataset. Both species had a positive association with the other species although prior studies suggest that the two species are rarely encountered simultaneously (Simon et al.2010). Negative relationship of one species to the other could have suggested potential avoidance behaviour. In areas where sympatric species occur, there is the potential for aggressive interactions, and porpoise deaths due to dolphin attack are widely reported in many areas, including Cardigan Bay (Jepson and Baker 1998; Deaville and Jepson 2006; Wilkin et al. 2012; Penrose 2014). Jacobson et al. (2015) reported clear partitioning and avoidance behaviour of porpoise when dolphins were present in Monterey Bay, California. Although no negative association was shown, trends in habitat use differ for the two species. It is possible that porpoises here may be avoiding areas and/or times at which dolphins are present to avoid aggressive interactions which could result in mortality. This may explain the niche partitioning observed. However, the pattern of detections could also simply reflect seasonal differences in habitat use rather than indicating avoidance behaviour, as disentangling effects of environmental factors from effects of dolphin presence is particularly difficult due the seasonality of occurrence of both species. At the same time, these environmental predictors of behaviour could obscure the real relationship between the two. The presence of the other species was found to be one of the least significant of the covariates in the statistical models (Table 2), and therefore the increase in harbour porpoise detections when bottlenose dolphins are not present may not only signify avoidance but also the natural variations in species occurrence in relation to preferred prey at sites in Cardigan Bay. It is possible that seasonal changes in the diet of the two species bring both into conflict with one another in this area, during times when the diet of the two overlaps most.

Static acoustic monitoring of cetaceans can be a powerful tool for assessing relative abundance, distribution and behaviour (Elliott et al. 2011; Kyhn et al. 2012; Nuuttila et al. 2013a), as cetaceans are only visible at the surface for between 1 and 20% of the time, making classification of animal activity based on their vocalisation more appropriate (Tyack and Miller 2002). However, all acoustic methods have some important limitations, not least the fact that it is only possible to detect individuals that are vocalising. Click loggers like the T-POD log echolocation clicks which are highly directional, and so only those vocalisations directed towards the recorder will have a chance of being detected. Furthermore, only animals echolocating within the detection range of the device will be logged, and this varies depending on environmental conditions, physical characteristics of the sea-bed, ambient noise, and amplitude and frequency of the emitted signals. Crucially, the two species have different maximum detection ranges as the source level of the dolphin echolocation can be much higher than that of the porpoise, and thus be detected from further away, up to several kilometres in some studies (Philpott et al. 2007; Nuuttila et al. 2013b) whereas the porpoise range has been found to be around few hundred meters (Kyhn et al. 2012). This discrepancy in sampling range could potentially bias the data by over emphasizing dolphin presence, although the effect of dolphins on porpoises, even if further away would probably be unaffected. In addition to issues of detectability, species classification can be problematic as dolphin echolocation signals can have energy within the porpoise frequency range. We attempted to minimise this by using only the high quality click trains for both species. Other studies which have investigated T-POD performance on dolphin detections have found that dolphins are generally well detected by the logger and included ‘Cet Lo’ (Bailey et al. 2010) and even ‘Doubtful’ Philpott et al. (2007) click trains categories in their analyses, although both conducted additional visual validation of their dolphin encounters. Here, the highest number of simultaneous detections was recorded during summer months when porpoise detections were at their lowest. If some of those porpoise detections were in fact dolphin detections, and the actual porpoise presence lower than reported here, the patterns of opposing habitat use and avoidance described in this paper would be even more evident. Future research is required to evaluate classification accuracy using static acoustic data loggers, especially as the train detection and classification algorithms of the T-POD software are proprietary and not available for user manipulation. There are some concerns over the comparability of data across regions and how differences in the sensitivity of different T-POD versions, different train algorithms, or even individual loggers affect the performance of the equipment and outcome of the analysis, especially when using the early versions. (Kyhn et al. 2008; Bailey et al. 2010). Here, an improved later version 4 was used although the variation in T-POD sensitivities may nevertheless have influenced the results. The T-POD has now been succeeded by the C-POD, which offers several improvements (Dähne et al. 2013; Nuuttila et al. 2013b). However, some T-PODs remain in use and continue to be an effective tool for cetacean monitoring (Bailey et al. 2010; Elliott et al. 2011; Leeney et al. 2011).

Despite its limitations, the T-POD and other similar acoustic devices provide an effective and cheap method for continuous, homogeneous data collection and its algorithm offers a rapid method of sampling otherwise enormous datasets. Yet acoustic detections do not indicate the true number of animals in the area, merely the number of vocalisations. For harbour porpoises, which echolocate almost continuously (Akamatsu et al. 2007; Linnenschmidt et al. 2013), and tend to occur solitarily or in groups of 2–3, this is likely to be a good reflection of their overall distribution between these sites, as well as indication of relative abundance. Bottlenose dolphins use echolocation mainly when feeding or travelling but less so when socialising and resting (Au 1993), so increased dolphin detection rates are likely to signify either increased movement around the monitoring site or increased foraging events (or both). However, a combined acoustic and visual study by Reyes-Zamudio (2005) found that dolphins at Mwnt and New Quay sites engaged in either foraging or travelling 91.8% of the time observed, showing that acoustic detections can be a good indication of their overall presence. On the other hand, they typically occur in groups, and these acoustic monitoring methods may be less good at describing their relative abundance.

A noteworthy finding is that harbour porpoise were logged at much higher rates than bottlenose dolphin, which was consistent between years, sites, and seasons. Harbour porpoises are often overlooked in visual surveys due to their small size, inconspicuous blow and undemonstrative surface behaviour; furthermore, visual surveys tend to mainly occur during summer months due to the additional challenges posed by trying to monitor this species during winter months (Simon et al. 2010). Importantly, porpoises were most commonly detected at night-time, highlighting a disadvantage of visual surveys for the species, which can only be conducted in daylight and in good weather. With these factors considered, it is possible this species has gone under-reported within the Cardigan Bay SAC, and that has a number of implications for conservation management of the species.

Harbour porpoises occur in coastal waters all around the UK (Reid et al. 2003). However, in UK waters there are currently no SACs designated specifically for the conservation of harbour porpoise. The Skerries and Causeway SAC in Northern Ireland, designated in 2012, lists the harbour porpoise as a qualifying feature, Grade C status, but not as the primary reason for selection (JNCC 2015), and currently this is the only site which has any form of management measures for the species. In Cardigan Bay SAC, harbour porpoise is only listed as an additional feature, with Grade D status of ‘insignificant presence’, and yet line transect surveys indicate a population of between two and three hundred individuals (Evans 2012; Feingold and Evans 2014). If animals are using the area predominantly nocturnally, the population here may be even higher. Three areas around Wales, namely the north of Anglesey, Cardigan Bay extending to the Pembrokeshire seas, and the outer Bristol Channel, are in the process of SAC designation, as they are considered to support internationally important populations of harbour porpoise (Natural Resources Wales 2015). The present study provides evidence that harbour porpoises occur regularly and throughout the year in Cardigan Bay SAC, meeting the criterion of ‘continuous or regular presence’ set out by the EU Habitats Directive (92/43/EEC 1992).

Marine mammals are susceptible to anthropogenic disturbances and dolphins. In Cardigan Bay SAC, the bulk of recreational vessel and commercial wildlife watching operations occurs through the summer months; fishing activities take place throughout the year, and scallop dredging is limited to winter months; therefore, it is likely that the two species face differing pressures from human activities. For large, mobile marine mammal species, it is not practical to designate their entire habitats as protected areas. Therefore, it is essential to identify those areas, which, if protected, would be most beneficial to the species’ survival, such as those used for feeding or breeding (Hoyt 2004). Without comprehensive acoustic monitoring data from outside the existing SAC, it is not possible to ascertain to what extent the current boundaries of Cardigan Bay SAC provide adequate protection for the two species but the data presented here does show the SAC to be a frequently used area by both, identifying seasonal, diel and tidal patterns of occurrence, demonstrating interspecific variation in abundance and distribution, and supporting visual evidence of high local abundance. Further research into porpoise and dolphin habitat use and abundance, including prey species analysis across Wales is required to understand what drives small cetacean presence in the region. This is a crucial step towards identifying areas of high importance for the harbour porpoise, especially with regards to species-specific management measures, as the protection afforded to bottlenose dolphins will not necessarily be adequate to protect porpoises given the differences in their habitat preference, seasonal presence, and fine scale temporal movements.

## Electronic supplementary material

Below is the link to the electronic supplementary material.

Supplementary material 1 (PDF 533 KB)
