# Supplementary material for: Don’t forget the porpoise: acoustic monitoring reveals fine scale temporal variation between bottlenose dolphin and harbour porpoise in Cardigan Bay SAC
Source: Mar Biol. 2017 Feb 21;164(3):50. doi: 10.1007/s00227-017-3081-5 (PMC5320002; doi:10.1007/s00227-017-3081-5)
Supplement: Supplementary file 1 — Supplementary material 1 (PDF 533 KB) [file 227_2017_3081_MOESM1_ESM.pdf]

Don't forget the porpoise: Acoustic monitoring reveals fine scale temporal variation between bottlenose dolphin and harbour porpoise in Cardigan Bay SAC

Nuuttila, Hanna K.[1,2]*, Courtene-Jones, Winnie[3], Baulch, Sarah[4], Simon, Malene[5,] and Evans, Peter G.H.[6] [1]

SEACAMS, Swansea University, Singleton Park, Swansea SA2 8PP, UK, [2] Sea Watch Foundation, Paragon House, New Quay, Ceredigion SA45 9NR, [3]Glebe House, Boncath, Pembrokeshire, SA37 0EN [4]University of York YO10 5DD, UK [5]Greenland Climate Research Center, Greenland Institute of Natural Resources, P.O. Box 570, Kivioq 2, 3900 Nuuk, Greenland, [6]Sea Watch Foundation, Ewyn y Don, Bull Bay, Amlwch, Isle of Anglesey LL68 9SD, UK

Electronic Supplement: GAM Summaries for final models

```
> summary(NBgamBND_LW_autoknots4all<-gam(HP ~ s(Month, k = 4,bs="cs")
+as.factor(Year)+s(Hour, k = 4,bs="cs") + s(Sunset_Diff, k=-1) + s(LW_Diff,k=4)
+ s(MaxTide, k=-1) + as.factor(HP.pres) + as.factor(Location), family=nb, data=Data))

Family: Negative Binomial(0.108)
Link function: log

Formula:
BND ~ s(Month, k = 4, bs = "cs") + as.factor(Year) + s(Hour,
    k = 4, bs = "cs") + s(Sunset_Diff, k = -1) + s(LW_Diff, k = 4) +
    s(MaxTide, k = -1) + as.factor(HP.pres) + as.factor(Location)

Parametric coefficients:
                          Estimate Std. Error z value Pr(>|z|)
(Intercept)               -1.08177    0.03419 -31.638   <2e-16 ***
as.factor(Year)2006       -0.46655    0.02741 -17.019   <2e-16 ***
as.factor(Year)2007       -2.65760    0.16697 -15.916   <2e-16 ***
as.factor(Year)2008       -0.51165    0.03106 -16.475   <2e-16 ***
as.factor(Year)2009       -0.60242    0.06703  -8.988   <2e-16 ***
as.factor(HP.pres)1        0.36010    0.02514  14.324   <2e-16 ***
as.factor(Location)AB_out  0.55448    0.04160  13.328   <2e-16 ***
as.factor(Location)CA     -1.60578    0.04750 -33.808   <2e-16 ***
as.factor(Location)CH_in  -2.42276    0.06518 -37.172   <2e-16 ***
as.factor(Location)CH_out -2.31840    0.07898 -29.354   <2e-16 ***
as.factor(Location)MW_in  -0.97698    0.04134 -23.631   <2e-16 ***
as.factor(Location)MW_out -0.77629    0.04598 -16.884   <2e-16 ***
as.factor(Location)NQ_fish 0.43737    0.03937  11.109   <2e-16 ***
as.factor(Location)NQ_reef -1.09942   0.04108 -26.763   <2e-16 ***
as.factor(Location)YN     -0.43138    0.03964 -10.883   <2e-16 ***
---
Signif. codes:  0 '***' 0.001 '**' 0.01 '*' 0.05 '.' 0.1 ' ' 1

Approximate significance of smooth terms:
                edf Ref.df  Chi.sq  p-value
s(Month)      2.998  3.000 6447.39  < 2e-16 ***
s(Hour)       2.945  3.000   74.14  < 2e-16 ***
s(Sunset_Diff) 6.140  7.365   44.06 3.87e-07 ***
s(LW_Diff)    2.868  2.987   18.04 0.000439 ***
s(MaxTide)    8.118  8.792  134.77  < 2e-16 ***
---
Signif. codes:  0 '***' 0.001 '**' 0.01 '*' 0.05 '.' 0.1 ' ' 1

R-sq.(adj) =  0.0533   Deviance explained = 29.1%
-REML =  73951  Scale est. = 1         n = 169620


> summary(NBgamHP_LW_autoknots4all<-gam(HP ~ s(Month, k = 4,bs="cs")
```

```
 +as.factor(Year)+s(Hour, k = 4,bs="cs") + s(Sunset_Diff, k=-1)
 + s(LW_Diff,k=4) + s(MaxTide, k=-1) + as.factor(BND.pres) +
as.factor(Location), family=nb, data=Data))

Family: Negative Binomial(0.177)
Link function: log

Formula:
HP ~ s(Month, k = 4, bs = "cs") + as.factor(Year) + s(Hour, k = 4,
    bs = "cs") + s(Sunset_Diff, k = -1) + s(LW_Diff, k = 4) +
    s(MaxTide, k = -1) + as.factor(BND.pres) + as.factor(Location)

Parametric coefficients:
                          Estimate Std. Error z value Pr(>|z|)
(Intercept)                0.04837    0.02351   2.057  0.03970 *
as.factor(Year)2006       -0.01356    0.01873  -0.724  0.46923
as.factor(Year)2007       -0.35778    0.04022  -8.896  < 2e-16 ***
as.factor(Year)2008        0.12720    0.02106   6.041 1.53e-09 ***
as.factor(Year)2009       -0.44841    0.03763 -11.918  < 2e-16 ***
as.factor(BND.pres)1       0.13978    0.02383   5.867 4.45e-09 ***
as.factor(Location)AB_out  0.43275    0.02939  14.725  < 2e-16 ***
as.factor(Location)CA     -0.64966    0.02879 -22.562  < 2e-16 ***
as.factor(Location)CH_in  -1.07860    0.03491 -30.900  < 2e-16 ***
as.factor(Location)CH_out -0.09702    0.03496  -2.775  0.00552 **
as.factor(Location)MW_in  -1.06500    0.02743 -38.827  < 2e-16 ***
as.factor(Location)MW_out -0.12564    0.03089  -4.067 4.75e-05 ***
as.factor(Location)NQ_fish 0.25601    0.02732   9.372  < 2e-16 ***
as.factor(Location)NQ_reef -1.03548   0.02690 -38.494  < 2e-16 ***
as.factor(Location)YN     -0.19953    0.02685  -7.432 1.07e-13 ***
---
Signif. codes:  0 ‘***’ 0.001 ‘**’ 0.01 ‘*’ 0.05 ‘.’ 0.1 ‘ ’ 1

Approximate significance of smooth terms:
               edf Ref.df  Chi.sq  p-value
s(Month)       2.996  3.000 6381.69  < 2e-16 ***
s(Hour)        2.927  3.000  209.08  < 2e-16 ***
s(Sunset_Diff) 6.312  7.512   42.54 6.38e-07 ***
s(LW_Diff)     2.976  3.000  109.75  < 2e-16 ***
s(MaxTide)     7.648  8.543  165.40  < 2e-16 ***
---
Signif. codes:  0 ‘***’ 0.001 ‘**’ 0.01 ‘*’ 0.05 ‘.’ 0.1 ‘ ’ 1

R-sq.(adj) =  0.0768   Deviance explained = 14.6%
-REML = 1.7711e+05  Scale est. = 1         n = 169620
```
